# RRP6 from *Trypanosoma brucei*: Crystal Structure of the Catalytic Domain, Association with EAP3 and Activity towards Structured and Non-Structured RNA Substrates

**DOI:** 10.1371/journal.pone.0089138

**Published:** 2014-02-18

**Authors:** Rosicler L. Barbosa, Pierre Legrand, Frank Wien, Blandine Pineau, Andrew Thompson, Beatriz G. Guimarães

**Affiliations:** Synchrotron SOLEIL, Gif-sur Yvette, France; University of Queensland, Australia

## Abstract

RRP6 is a 3′–5′ exoribonuclease associated to the eukaryotic exosome, a multiprotein complex essential for various RNA processing and degradation pathways. In *Trypanosoma brucei*, RRP6 associates with the exosome in stoichiometric amounts and was localized in both cytoplasm and nucleus, in contrast to yeast Rrp6 which is exclusively nuclear. Here we report the biochemical and structural characterization of *T. brucei* RRP6 (*Tb*RRP6) and its interaction with the so-called *T. brucei* Exosome Associated Protein 3 (*Tb*EAP3), a potential orthologue of the yeast Rrp6 interacting protein, Rrp47. Recombinant *Tb*EAP3 is a thermo stable homodimer in solution, however it forms a heterodimeric complex with *Tb*RRP6 with 1∶1 stoichiometry. The crystallographic structure of the *Tb*RRP6 catalytic core exposes for the first time the native catalytic site of this RNase and also reveals a disulfide bond linking two helices of the HRDC domain. RNA degradation assays show the distributive exoribonuclease activity of *Tb*RRP6 and novel findings regarding the structural range of its RNA substrates. *Tb*RRP6 was able to degrade single and double-stranded RNAs and also RNA substrates containing stem-loops including those with 3′ stem-loop lacking single-stranded extensions. Finally, association with *Tb*EAP3 did not significantly interfere with the *Tb*RRP6 catalytic activity *in vitro*.

## Introduction

Kinetoplastids are flagellated protists many of them pathogenic to men and domestic animals. The most studied members of the class are the trypanosomes and leishmanias, which cause a number of serious diseases. For example, *Leishmania* species cause different types of infections regarded as cutaneous, mucocutaneous or visceral leishmaniasis in tropical countries around the world. *Trypanosoma brucei* is the causative agent of sleeping sickness in humans and nagana disease in cattle in sub-Saharan Africa while a related species, *T. cruzi*, causes Chagas disease in Latin America. These protozoa are unique in the sense that Kinetoplastidae genomes are deficient in regulatory transcription factors with control of gene expression, relying almost exclusively on post-transcriptional mechanisms (reviewed in [1]–[4]). Thus, a major mechanism available for kinetoplastids to regulate gene expression involves control of RNA processing and degradation rates.

Most of the enzymes involved in RNA metabolism in yeast and mammals have orthologues in trypanosomes and leishmanias, such as the exosome complex, although significant regulatory and biochemical differences are expected to be found given the central role of RNA stability control for gene expression in Trypanosomatids. The exosome is a 3′–5′ exoribonuclease complex that plays a central role in numerous pathways related to RNA processing and degradation, both in the nucleus and in the cytoplasm. Initially described in yeast [5], exosomes are found in archaea and eukaryotes from protozoa to mammals. The eukaryotic exosome core is constituted by nine subunits (Exo-9), structurally organized in a ring of three heterodimers formed by RNase PH-related proteins. This hexameric ring is capped by three subunits with homology to KH and S1 RNA-binding domains [6], [7]. In yeast and human cells the Exo-9 core is devoid of catalytic activity, the exosome ribonuclease activity is provided by the association of two nucleases, Rrp44 (also known as Dis3) and Rrp6 [6], [8]. Rrp44, the exosome tenth subunit, interacts with Exo-9 in the nucleus and cytoplasm and is essential for cell viability [5], [9]. It presents endonuclease and processive hydrolytic 3′–5′ exonuclease activities [6], [10], [11], which are modulated by the interaction with the Exo-9 core [12], [13]. In yeast, crystallographic and biochemical studies have shown that the Exo-9 central channel directs the RNA substrate to degradation by Rrp44 which is located on the opposite side of the S1/HK subunits [7], [12].

The eleventh exosome subunit, RRP6, is a member of the DEDD superfamily (DEDD-Y subgroup) of divalent metal dependent exonucleases [14], [15]. In yeast, Rrp6 is found exclusively in the nuclear exosome, whereas in human, RRP6 is more concentrated in the nucleoli but is also found, at lower concentration, associated to the nucleoplasmic and the cytoplasmic exosome [16]. Rrp6 is not essential for cell viability but deletion of the gene in yeast causes slow growth and high temperature sensitive phenotypes and accumulation of extended forms of 5.8S and snoRNAs [17], [18]. Interaction of Rrp6 with the exosome stimulates both Rrp44 exo and endoribonuclease activities. On the other hand, the Rrp6 catalytic activity is inhibited by a mutation in the Rrp44 exoribonuclease active site of the Exo-11 complex [13]. The crystal structure of the yeast Exo-10 bound to an RNA substrate and the C-terminal region of Rrp6 evidenced that Rrp6 indirectly stabilizes the complex exosome-RNA without a direct contact with the RNA [7].

Rrp6 is composed of an N-terminal PMCN2NT domain which was shown to interact with a cofactor Rrp47 [19], the EXO domain containing the catalytic active site, an HRDC (helicase and RNAseD C-terminal) domain, and a C-terminal region responsible for the interaction with the exosome [7], [18]. The crystal structures of the catalytic core of yeast and human RRP6 have shown the structural organization of the EXO and HRDC domains and revealed the conformation of the RRP6 active site residues in the presence of metals and/or nucleotides [20], [21]. RNA degradation assays have shown that human RRP6 is more efficient to degrade structured RNA substrates in comparison with yeast Rrp6, and the accessibility to the active site was proposed to play a role in this substrate selectivity [21].

The Rrp6 cofactor in yeast, Rrp47, and its human orthologue C1D are nuclear exosome-associated proteins that bind both RNA and DNA molecules with an apparent specificity for double-stranded DNA and structured RNA substrates [19], [22]–[24]. Rrp47/C1D proteins are conserved throughout eukaryotes and they are composed of a Sas10/C1D domain (*Pfam: protein families data base* domain PF04000) in the N-terminal region, and a more variable C-terminal. Genetic complementation assays identified the Sas10/C1D domain of Rrp47 as critical for yeast normal growth, sufficient for Rrp47 function *in vivo* and responsible for the interaction with the N-terminal domain of Rrp6. However, stable binding of Rrp47 to RNA *in vitro* requires both N-terminal and C-terminal regions. The C-terminal of Rrp47 was also shown to be involved in snoRNA maturation [24].

The characterization of the exosome complex in *Trypanosoma brucei* showed that its composition is similar to the yeast and human counterparts, being composed of six RNase PH-related proteins (*Tb*RRP41A, *Tb*RRP41B, *Tb*RRP45, *Tb*EAP1, *Tb*EAP2 and *Tb*EAP4) and three subunits related to the S1 domain proteins (*Tb*RRP4, *Tb*RRP40 and *Tb*CSL4). The RRP6 subunit, which is specific to the nuclear exosome in yeast, is localized in both nucleus and cytoplasm in *T. brucei* [25]–[27]. In contrast, Rrp44-like protein was not detected in *T. brucei* purified exosome fractions [26]. An additional subunit, *Tb*EAP3 (for Exosome Associated Protein 3), which resembles yeast Rrp47, was identified and its interaction with *Tb*RRP6 was detected by two-hybrid analysis [26]. The structural characterization of the native exosome from *Leishmania tarentolae* by electron microscopy revealed a molecular envelope which is consistent with the Exo-9 structure, and evidenced an additional density at the top of the Exo-9 core that could account for the *Tb*RRP6 and *Tb*EAP3 associated proteins [28]. Surprisingly, the purified *L. tarentolae* exosome, containing the RRP6 protein, showed no RNAse D-like activity *in vitro* [28].

Despite the amount of data presently available on the exosome complex, structural and functional information about the subunit RRP6 and its cofactor Rrp47 are still lacking. We have been especially interested in the study of their orthologues in kinetoplastids, motivated by the differences in the composition and activity of the trypanosome exosome regarding the subunits associated to the Exo-9 core. To better investigate the catalytic activity of *T. brucei* RRP6 and its interaction with EAP3 we have produced recombinant constructs of both proteins and performed structural studies and degradation assays against different RNA substrates.

## Materials and Methods

### Cloning and Site-direct Mutagenesis

**Figure 1:**
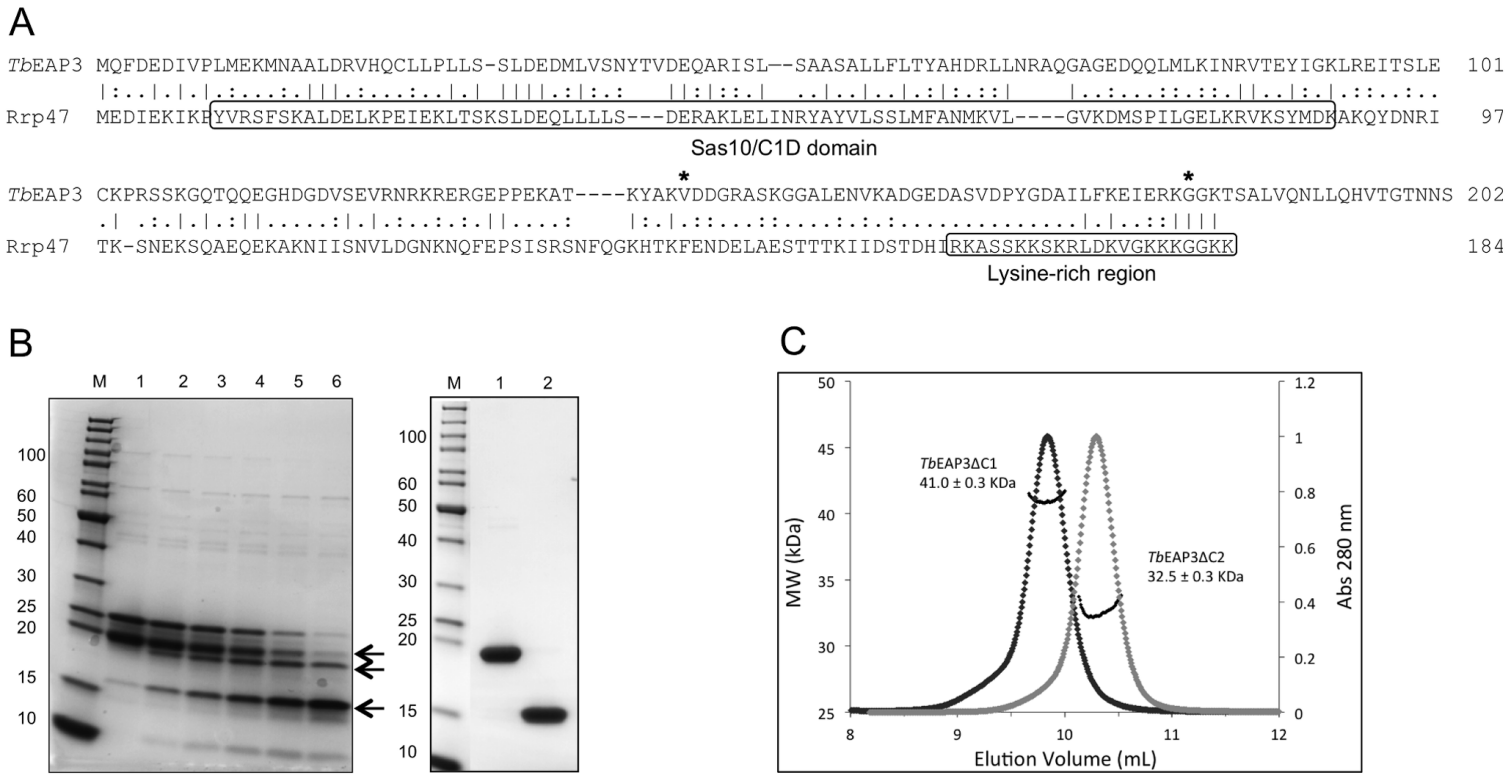
Primary structure analysis and purification of *T. brucei* EAP3. A) Sequence alignment of *Tb*EAP3 protein and yeast Rrp47 using the EMBOSS Needle program for pairwise sequence alignment. The Sas10/C1D domain and the lysine-rich region of Rrp47 are highlighted in boxes. The asterisks indicate the last residues of the two C-terminal deletion mutants *Tb*EAP3ΔC1 (aa 1–183) and *Tb*EAP3ΔC2 (aa 1–144), produced in this work. B) Left, SDS-PAGE analysis of *Tb*EAP3 limited proteolysis assay; M: molecular marker (sizes in kDa are shown on the right); lane 1: control without protease; lanes 2 to 6: chymotrypsin digestion patterns after 15, 30, 60, 120 and 210 minutes. The bands corresponding to the main fragments resulting by limited proteolysis are indicated by arrows. Right, SDS-PAGE analyses of *Tb*EAP3ΔC1 and *Tb*EAP3ΔC2 mutants after purification. C) SEC-MALS analyses indicate that *Tb*EAP3ΔC1 and *Tb*EAP3ΔC2 are homodimers in solution.

**Figure 2:**
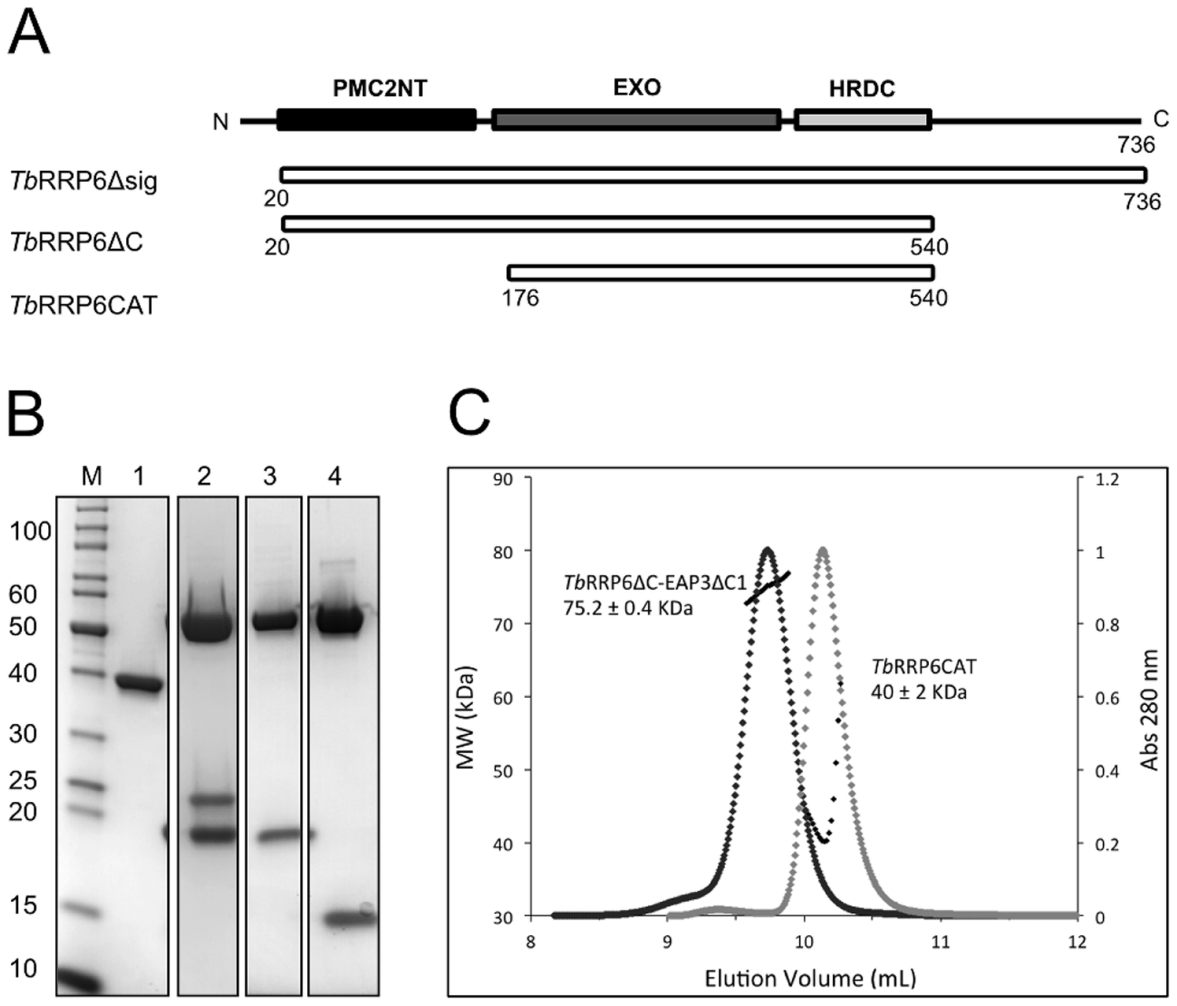
Purification of *Tb*RRP6 constructions and complexes with *Tb*EAP3. A) Schematic representation of the *Tb*RRP6 constructions tested for recombinant expression. *Tb*RRP6CAT includes the EXO and HRDC domains that constitute the catalytic core of the protein; *Tb*RRP6ΔC, in addition to the EXO and HRDC, contains the N-terminal PMC2NT domain, which was shown to interact with Rrp47 in yeast [19]. *Tb*RRP6Δsig comprises the entire protein except for a 19-residues N-terminal sequence identified as a signal peptide. B) SDS-PAGE analysis of the purified *Tb*RRP6CAT (lane 1) and the co-purified complexes *Tb*RRP6ΔC-EAP3, *Tb*RRP6ΔC-EAP3ΔC1 and *Tb*RRP6ΔC-EAP3ΔC2 (lanes 2, 3 and 4, respectively). The additional band observed during the purification of the complex *Tb*RRP6ΔC-EAP3 (lane 2) corresponds to the degradation product of *Tb*EAP3. The sizes of the molecular weight markers (M) are given in kDa. C) SEC-MALS analyses of *Tb*RRP6CAT and the complex *Tb*RRP6ΔC-EAP3ΔC1. The estimated molecular masses indicate that *Tb*RRP6CAT is a monomer in solution while *Tb*RRP6ΔC and *Tb*EAP3ΔC1 form a heterodimer.

The genes encoding *Tb*RRP6 (Tb927.4.1630) and *Tb*EAP3 (Tb927.7.5460) were synthesized by GeneArt Gene Synthesis (Life Technologies). The corresponding *Tb*RRP6 DNA sequences were subcloned into the pET28a vector (Novagen) to express *Tb*RRP6Δsig (residues 20–736), *Tb*RRP6ΔC (residues 20 to 540) and *Tb*RRP6CAT (residues 176 to 540) constructs in fusion with a C-terminal His-tag. *Tb*EAP3 coding sequence was subcloned into the pET21a vector (Novagen) to express the full-length *Tb*EAP3 protein and the truncated forms *Tb*EAP3ΔC1 (residues 1 to 183) and *Tb*EAP3ΔC2 (residues 1 to 144) (Figures 1A and 2A).

Site-direct mutagenesis was performed in *Tb*RRP6CAT and *Tb*RRP6ΔC constructs using the GeneArt Site-Directed Mutagenesis System (Life Technologies) in order to introduce two single-point mutations in the catalytic site, converting D271 to N and Y393 to A; and two single-point mutations in the HRDC domain, converting both C496 and C515 to S. Gene amplification and bacterial transformation followed the protocol described by the manufacturer and the mutated DNA sequences were screened by PCR. The presence of mutations was confirmed by nucleotide sequencing. Table S1 shows the list of the primers used to generate the mutations.

### Protein Expression and Purification

The same general protocol was used for expression and purification of all proteins/complexes studied in this work. Specific differences are described when appropriated. *Escherichia coli* cells C3022 (New England Biolabs) were transformed or co-transformed with the pET expression vectors. The cultures were grown at 37°C in LB media containing ampicillin 100 µg/mL or kanamycin 50 µg/mL, for the clones on pET21a and pET28a, respectively; whereas both antibiotics were used for co-expression of the proteins in the complexes *Tb*RRP6ΔC-EAP3ΔC1 and *Tb*RRP6ΔC-EAP3ΔC2. When the culture reached the OD_600_ ∼ 0.6, the temperature was reduced to 18°C and protein expression was induced with 0.3 mM of isopropyl-β-D-thiogalactopyranoside (IPTG), overnight. Cells were harvested by centrifugation at 4000 g for 20 minutes and the pellet was stored at −80°C until protein purification.

The frozen cells from 1 L culture were suspended and lysed in 30 mL of buffer A (50 mM Tris-HCl pH 8.0, 300 mM NaCl, 15 mM Imidazole, 5% glycerol) containing protease inhibitor cocktail (Roche Applied Science) and 1 mg/mL of lysozyme. After incubation of 30 minutes at 4°C, benzonase (25 units/mL) was added to the suspension. Cell extracts were isolated by sonication and centrifugation at 40000 g for 30 minutes at 4°C. The extracts were loaded onto a 5 mL His-Trap FF column (GE Healthcare Life Sciences) equilibrated in buffer A. The proteins were eluted with a 10 column volumes linear gradient from 0 to 400 mM of imidazole in the same buffer. Fractions containing the target proteins were pooled, concentrated and loaded onto a Superdex 200 16/60 column (GE Healthcare Life Sciences) equilibrated with 50 mM Tris-HCl pH 8.0, 500 mM NaCl, 5% glycerol. During the size exclusion chromatography step, both *Tb*EAP3ΔC1 and *Tb*EAP3ΔC2 proteins were eluted in two major well resolved peaks, one of them corresponding to aggregates. Only the non-aggregated fractions were used in the next experiments. Fractions containing the target proteins were pooled and dialyzed against 50 mM Tris-HCl pH 8.0, 10 mM NaCl, 5% glycerol for salt removal before loading onto an anion exchange column. The last purification step was performed using a mono Q 5/50 GL column (GE Healthcare Life Sciences) equilibrated with the same buffer utilized in the dialysis. The proteins were eluted in a linear NaCl gradient (from 10 mM to 1 M) and the final pool of eluted fractions was dialyzed against 50 mM Tris-HCl pH 8.0, 50 mM NaCl, 5% glycerol and concentrated using an Amicon Ultra centrifugal filter to approximately 10 mg/mL.

### Limited Proteolysis and Mass Spectrometry Analysis

*Tb*EAP3 was submitted to limited proteolysis in an attempt to identify stable fragments. After testing different proteases and *Tb*EAP3 concentrations, best results were obtained with chymotrypsin at a ratio of 1∶1000 (w/w) of protease to *Tb*EAP3. Digestion reactions were carried out at 20°C in buffer containing 100 mM Tris-HCl pH 7.5, 100 mM NaCl and 1 mM β-mercaptoethanol. Samples were taken after incubation times of 15, 30, 60, 120 and 210 minutes, and the reactions stopped by heating at 95°C into the Laemmli loading buffer. Limited proteolysis results were analyzed by SDS-PAGE. Electrophoretic bands were sliced out of the gel and submitted to MALDI mass spectrometry analysis at the SICPaPS facility of the Imagif-CNRS platform, Gif sur Yvette, France.

### Size Exclusion Chromatography Combined with Multi-Angle Light Scattering (SEC-MALS)

The SEC-MALS technique was employed to determine de molecular weight of the proteins *Tb*EAP3ΔC1, *Tb*EAP3ΔC2, *Tb*RRP6CAT and the complexes *Tb*RRP6ΔC-EAP3ΔC1 and *Tb*RRP6ΔC-EAP3ΔC2 in solution. The measurements were performed at the Biophysics facility of the Imagif-CNRS platform, Gif-sur Yvette, France. Size exclusion chromatography (SEC) experiments were performed using an HPLC system (Shimadzu) with the proteins concentrated at 5 mg/mL in buffer 50 mM Tris-HCl pH 7.5, containing 500 mM NaCl and 5% glycerol. The columns were previously equilibrated with the same buffer and sample loading and elution were performed at a flux of 0.5 mL/min. The column KW-804 (Shodex) was used for the analyses of the complex *Tb*RRP6ΔC-EAP3ΔC1 and the *Tb*EAP3 constructs and the column KW-803 (Shodex) was used for the analyses of *Tb*RRP6CAT and the complex *Tb*RRP6ΔC-EAP3ΔC2. Coupled to the size exclusion chromatography, multi-angle light scattering (MALS) measurements were performed using a miniDAWN TREOS system (Wyatt Technology), to determine the absolute molar mass at each retention volume.

### RNA Degradation Assays

The 5′-end fluorescein (FAM) labeled RNA oligonucleotides were purchased from Eurogentec. For degradation assays on single and double-stranded substrates, the RNA sequence 5′- FAM-CCAAAAAAAAAAAAACCACUCACUCACUCA with or without the complementary RNA strand (called ssRNA and dsRNA, respectively) was used. In addition, five RNA AU-rich oligonucleotides were synthesized containing a GNRA sequence designed to introduce a structured stem-loop in different positions of the chain. The substrates were named GNRA0 when the stem-loop is present at the 3′-end, and GNRA5, GNRA20, GNRA24 or GNRA29 when the substrates contain a 3′ non-structured extension of 5, 20, 24 or 29 nucleotides, respectively. The sequences are shown below:

GNRA0∶ 5′FAM-AUUUAUUAUUAUUUAUUUAUUAUUUAUUAGGGCGGGCGCAAGCCCGCCC 3′

GNRA5∶ 5′FAM-UUAUUAUUUAUUUAUUAUUUAUUAGGGCGGGCGCAAGCCCGCCCAUUUA 3′

GNRA20∶ 5′FAM-AUUUAUUUAGGGCGGGCGCAAGCCCGCCCUAUUUAUUAUUUAUUAUUUA 3′

GNRA24∶ 5′FAM-AUUUAGGGCGGGCGCAAGCCCGCCCAUUAUUUAUUAUUUAUUAUUUAUU 3′

GNRA29∶ 5′FAM-GGGCGGGCGCAAGCCCGCCCAUUAUUUAUUAUUUAUUAUUUAUUAUUUA 3′

All substrates were heated at 85°C and cooled down to room temperature before each assay. Single-point reactions were performed using 0.1 µM RNA and 0.1, 0.5 or 1 µM of protein. Reactions lasted for 40 minutes at 20°C or 37°C in buffer 10 mM Tris-HCl (pH 8.0, 7.5, 7.0 or 6.5) containing 50 mM KCl, 10 mM DTT, 1 u/µL of RNase inhibitor and 5 mM MnCl_2_ or MgCl_2_. Time-course reactions were performed at 37°C in the mixture described above with 10 mM Tris-HCl pH 8.0, 5 mM MnCl_2_ and 0.1 µM of protein. Reactions were stopped after incubation times of 0, 1, 3, 5, 10, 20 and 40 minutes by addition of buffer containing 95% formamide, 18 mM EDTA, 0.025% SDS, xyleno cyanol and bromophenol blue and heating at 95°C during 5 minutes. Samples were loaded onto a 15% polyacrylamide-8 M urea gel using 1X TBE buffer. The gels were visualized using a Fujifilm image LAS-3000 with the emission and detection wavelengths set at 460 nm and 510 nm, respectively, and exposure time of 20 seconds.

### Electrophoretic Mobility Shift Assay (EMSA)

The oligonucleotides used in EMSAs were obtained from Eurogentec and their sequences are listed below:

RNA: 5′ FAM-CCAAAAAAAAAAAAACCACUCACUCACUCA 3′

DNA: 5′ FAM-CCAAAAAAAAAAAAACCACTCACTCACTCA 3′

RNArev: 5′ UGAGUGAGUGAGUGGUUUUUUUUUUUUUGG

DNArev: 5′ TGAGTGAGTGAGTGGTTTTTTTTTTTTTGG

RNA and DNA containing a 5′ fluorescein label (FAM), were used as single-stranded substrates (ssRNA and ssDNA) or combined with RNArev and DNArev to generate double-stranded probes (dsRNA and dsDNA). Before each assay, the oligonucleotides (50 nM) were heated at 85°C for 3 minutes and then cooled to room temperature. Binding reactions lasted for 30 minutes on ice in buffer 10 mM Tris-HCl pH 8.0 containing 10 mM DTT, 25 mM KCl and 5 mM MnCl_2_ after addition of the protein at 5, 10 or 50 µM. Reaction products were resolved on native 6% acrylamide gels equilibrated in running buffer containing 0.25X TBE and 0.5% glycerol on ice. The gels were visualized using a Fuji LAS-3000 imager with the emission and detection wavelengths set at 460 nm and 510 nm, respectively, and exposure time of 20 seconds.

### Synchrotron Radiation Circular Dichroism (SRCD)

Synchrotron radiation circular dichroism (SRCD) experiments, covering the UV spectral range from 260–170 nm, were conducted at the DISCO beam line of the Synchrotron SOLEIL [29], [30]. The use of SRCD allowed us to extend the spectral range and improve the signal to noise ratio obtained (as compared to conventional CD [31]) in the far UV, with peaks down to 190 nm clearly identifiable. The proteins *Tb*RRP6CAT (200 µM), *Tb*EAP3ΔC1 (300 µM), *Tb*EAP3ΔC2 (500 µM) and the complexes *Tb*RRP6ΔC-EAP3ΔC1 (100 µM) and *Tb*RRP6ΔC-EAP3ΔC2 (100 µM) were dialyzed against 100 mM sodium phosphate buffer pH 8.0. Protein concentrations were calculated from their extinction coefficients [32] and the measured absorption at 280 nm (in triplets) using a nanodrop spectrophotometer (ThermoFisher). Temperature scans were performed between 18°C and 78°C in 3°C steps with 2 minutes settling time. All spectra were acquired using a CaF_2_ 12 µm optical path cell [33]. For each measurement, the mean value of three spectra was calculated before subtraction of the baseline and zeroing between 263–270 nm. Spectra were calibrated to a standard solution of (+)-camphour-10-sulphonic acid (CSA), and normalized and converted to Δε using the software CDtool [34]. The secondary structure content was determined with the DichroWeb interface [35] using SELCON3 and CONTIN methods, with the reference set SP175 and a spectral cutoff at half of the total high tension variation of the photomultiplier [31]. The thermal denaturation curves were calculated at 222 nm, and the melting temperatures (T_m_) were determined from a sigmoidal fit. When required, the proteins’ spectra were collected in the presence of 5 mM of MgCl_2_ or 10 mM of DTT to evaluate their effect on the protein stability.

### Crystallization, Diffraction Data Collection and Processing

Extensive crystallization trials were performed using commercial screens with all proteins and complexes produced and purified to homogeneity. Hanging and/or sitting drop vapor-diffusion methods were tested at different protein concentrations. Promising hits were obtained only for the *Tb*RRP6CAT constructs. Optimization of the crystallization conditions were performed by varying pH, precipitant and protein concentrations. Sea urchin-like crystals were obtained by hanging drop vapor diffusion at 18°C by mixing the *Tb*RRP6CAT protein at 10 mg/mL in 50 mM Tris-HCl pH 8.0, 250 mM NaCl, 5% glycerol with crystallization buffer containing 0.1 M Tris-HCl pH 7.5 and 26% (v/v) PEG 3350. Before data collection at cryogenic temperature, the crystals were cryoprotected with 20% PEG 400 added to the mother liquor prior to flash-cooling in liquid nitrogen. Crystals of the mutant *Tb*RRP6CAT-C496S (9 mg/mL in 50 mM Tris-HCl pH8.0, 50 mM NaCl, 5% glycerol) were obtained by the sitting drop vapor diffusion method after mixing the protein and the well solution containing 25% PEG 2000. The crystals were cryoprotected by adding 25% PEG 400 to the mother liquor. X-ray diffraction data were collected from needle-shaped crystals of approximately 50 µm length and 5 µm width at the PROXIMA 1 beam line of the Synchrotron SOLEIL using a PILATUS 6 M detector. The diffraction data were processed with the XDS package [36]. *Tb*RRP6CAT and *Tb*RRP6CAT-C496S crystals belong to space group P2_1_ and diffracted to 2.40 Å and 2.15 Å resolution, respectively. Data collection statistics are reported in Table 1.

**Table 1:** Crystallographic data and refinement statistics.

	*Tb*RRP6CAT	*Tb*RRP6CAT-C496S
*Data statistics*		
Source	SOLEIL-PX1	SOLEIL-PX1
Wavelength (Å)	0.9801	0.9801
Resolution (Å)	50–2.4 (2.54–2.40)	50–2.15 (2.28–2.15)
Space group	P2_1_	P2_1_
Unit cell (Å)	39.5, 92.7, 49.9/β = 104.8	39.4, 93.4, 49.9/β = 105.2
Number of observations	39831 (4851)	71910 (10744)
Number of unique reflections	13174 (1877)	19025 (3074)
Completeness	95.9 (85.9)	99.5 (98.5)
Redundancy	3.0 (2.6)	3.7 (3.5)
Mean I/lδl	10.21 (2.8)	9.13 (1.6)
Rmeas (%)	10.3 (45.3)	13.5 (89.6)
*Refinement statistics*		
R_work_/R_free_	0.16/0.22	0.17/0.22
Bond RMSD length (Å)/angle (°)	0.01/1.1	0.01/1.1
Average B-protein/water (Å^2^)	36.1/38.9	38.1/44.0
Non-hydrogen atoms (except waters)	2866	2854
Number of water molecules	185	212
*Ramachandran plot (Molprobity)*		
Favored (%)	98.3	98.6
Outliers (%)	0	0
*PDB code*	4NLB	4NLC

Values in parenthesis correspond to the outer resolution shell.

### Structure Resolution and Refinement

The structure of *Tb*RRP6CAT was solved by molecular replacement with the program PHASER [37] using the atomic coordinates of the human RRP6 (PDB code 3SAF) [21] as the search model. Rigid body refinement using the model of the native *Tb*RRP6CAT was applied in order to obtain the initial electron density map for the mutant *Tb*RRP6CAT-C496S. Refinement of the structures were performed alternating cycles of BUSTER [38] with visual inspection and manual rebuilding using COOT [39]. For *Tb*RRP6CAT, a total of 359 residues, out of the 365 expected excluding the His-tag fusion, were modeled for one monomer in the asymmetric unit. 185 water molecules were added during the refinement cycles and the R_factor_/R_free_ values converged to 0.16/0.22. For the mutant *Tb*RRP6CAT-C496S, 355 residues and 212 water molecules were modeled in the asymmetric unit and the R_factor_/R_free_ values converged to 0.17/0.22. The stereochemistry of the models was analyzed with MolProbity [40] and no outliers were observed in the Ramachandran plot. Refinement statistics are summarized in the Table 1. Electrostatic potential were calculated using the Adaptive Poisson-Boltzmann Solver [41] through the PDB2PQR Server [42].

## Results

### Recombinant *Tb*EAP3 is a Thermo Stable Homodimer in Solution

EAP3 was identified as a component of the *T. brucei* exosome which interacts with the conserved subunit RRP6 [26]. Position-specific iterated BLAST search using the sequence of *Tb*EAP3 against a non-redundant data base which excludes Trypanosomatidae proteins results only in poor hits and does not detect the yeast orthologue Rrp47, a small nuclear protein known to interact with Rrp6 and involved in RNA maturation [24]. However, the sequence alignment of *Tb*EAP3 and yeast Rrp47 using the EMBOSS Needle program for pairwise alignment (available through the EMBL-EBI server; http://www.ebi.ac.uk) showed that *Tb*EAP3 primary structure shares 21% identity with Rrp47. Although the major sequence similarity is found in the N-terminal region, the bioinformatically predicted Sas10/C1D domain, present in Rrp47 and proposed to represent a group of nucleic acid binding proteins, is not detected when the *Tb*EAP3 sequence is analyzed for *Pfam* matches (protein families’ database; http://pfam.sanger.ac.uk). Moreover, several residues of the Rrp47 lysine-rich region, which is required for RNA interaction [24] are not conserved in *Tb*EAP3 which also presents a C-terminal extension of 17 residues relative to Rrp47 (Figure 1A).

To gain insights into the *Tb*EAP3 function and its ability to interact with RRP6 we expressed and purified the recombinant *Tb*EAP3 in *E. coli* cells. The recombinant protein is unstable in solution. Degradation products were observed early after cell disruption and throughout the purification process. In order to identify the protease-sensitive regions of *Tb*EAP3, limited proteolysis experiments were performed which evidenced two main stable fragments (Figure 1B, left). These fragments were identified by mass spectrometry (data not shown). Based on the mass spectrometry results, two new constructs were designed to express *Tb*EAP3ΔC1 and *Tb*EAP3ΔC2 C-terminal truncated proteins (Figure 1A). Both variants were expressed in *E. coli* cells and purified to homogeneity (Figure 1B, right).

Previous size exclusion chromatography assays have described yeast Rrp47 as a hexamer in solution [19]. Based on analytical gel filtration chromatography, the calculated molecular mass of *Tb*EAP3 is compatible with the size of a trimer (data not shown). To confirm this result, we submitted both *Tb*EAP3ΔC1 and *Tb*EAP3ΔC2 variants to size exclusion chromatography combined with multi-angle light scattering (SEC-MALS) analysis. SEC-MALS results showed unique monodisperse peaks for *Tb*EAP3ΔC1 and *Tb*EAP3ΔC2 with molecular masses estimated in 41.0 kDa and 32.5 kDa, respectively, which correspond to homodimers in solution (Figure 1C). In addition, mass spectrometry analysis also revealed a dimer of *Tb*EAP3ΔC1 (measured mass of 42.5 kDa) under denaturing conditions and indicated the presence of an intermolecular disulfide bond (data not shown). The inaccuracy of the initial results obtained for *Tb*EAP3 by analytical gel filtration chromatography may be explained by the presence of large flexible/unfolded regions, such as those susceptible to limited proteolysis, that increase the hydrodynamic radius of the protein. The C-terminally truncated constructs, however, show the expected molecular mass for homodimers. These findings are in agreement with recent data reported for yeast Rrp47, showing by analytical ultracentrifugation, that it is also purified as a homodimer [43].

Secondary structure content and thermal stability of *Tb*EAP3ΔC1 and *Tb*EAP3ΔC2 were determined using SRCD. CD spectra were measured within a wavelength range of 170 to 260 nm and data analyses show a secondary structure content estimate of 36% and 41% of alpha-helix, 15% of beta-sheet and 49% and 44% of random structures for *Tb*EAP3ΔC1 and *Tb*EAP3ΔC2, respectively (Table 2). The higher content of random structures found in *Tb*EAP3ΔC1 indicates that the C-terminal region of *Tb*EAP3 might be unfolded. The mutants were then submitted to thermal denaturation and the melting temperatures were estimated by analysis of the peaks at 222 nm, indicating a structural stability up to around 55°C for both constructs. SRCD results are summarized in Table 2 and the thermal denaturation spectra of the *Tb*EAP3ΔC1 variant is shown in the supporting information (Figure S1). To investigate the possible role of an intermolecular disulfide bond in the stabilization of the *Tb*EAP3 dimer, we performed SEC-MALS experiments and thermal denaturation under highly reducing conditions. In the presence of 10 mM DTT, CD analyses revealed no changes in the secondary structure content estimated at 20°C (Table 2), although the melting temperatures of both variants decreased by at least 10°C. Moreover, SEC-MALS analysis showed that *Tb*EAP3ΔC1 and *Tb*EAP3ΔC2 keep the dimeric structure in solution under reducing condition (data not shown). These results indicate that the rupture of the intermolecular disulfide bond affects *Tb*EAP3 thermal stability but the quaternary structure is maintained by non-covalent interactions. Interestingly, yeast Rrp47, which does not contain any cysteine residue in its sequence, was also described as a homodimer in solution [43].

**Table 2:** Secondary structures content and melting temperatures of the target proteins based on SRCD measurements.

Sample	% α-helix	% β-sheet	% turn	% others	RMSD	T_m_ 222 nm (°C)
**EAP3ΔC1**	36	15	17	32	0.04	55±2
**EAP3ΔC1/DTT**	36	16	16	32	0.07	44±1
**EAP3ΔC2**	41	15	14	30	0.06	55±1
**EAP3ΔC2/DTT**	39	18	13	30	0.02	42±2
**RRP6CAT**	39	19	11	31	0.08	35.2±0.2
**RRP6ΔC-EAP3ΔC1**	45	14	11	30	0.04	50.6±0.3
**RRP6ΔC-EAP3ΔC2**	48	13	11	28	0.06	40.0±0.6

RMSD is the root-mean-square deviation between the experimental and the calculated spectra. The melting temperatures (T_m_) were estimated based on thermal denaturation curves calculated at 222 nm. When indicated the samples were treated with 10 mM of DTT prior to the measurements.

Previous studies performed with yeast Rrp47 showed its ability to bind double-stranded DNA and structured RNA but not single-stranded or poly(A) substrates [19]. The human Rrp47 orthologue was shown to interact with tRNA and poly(G) but not poly(A), poly(C) or poly(U) [23]. We performed electrophoretic mobility shift assays to test the binding of the EAP3 variants *Tb*EAP3ΔC1 and *Tb*EAP3ΔC2 to single and double-stranded RNA and DNA substrates and to a stem-loop containing RNA (GNRA20). We were not able to detect protein-RNA or DNA interaction for any of these protein-substrate combinations, despite several attempts to optimize the experimental conditions. Regarding a possible conserved function, the experiments with Rrp47 truncated mutants, evidencing that the C-terminal lysine-rich region is essential for its RNA binding activity *in vitro* [24], are consistent with the lack of interaction of the *Tb*EAP3ΔC1 and *Tb*EAP3ΔC2 truncated proteins with RNA. Unfortunately, we were not able to confirm whether the C-terminal of *T. brucei* EAP3 mediates interactions with RNA since the intact protein could not be purified.

### *Tb*RRP6 forms a Heterodimer with *Tb*EAP3

To study the *T. brucei* RRP6 activity and its interaction with *Tb*EAP3 we initially worked with three *Tb*RRP6 constructs. *Tb*RRP6Δsig (residues 20–736) is the largest construct and lacks the N-terminal residues predicted to act as a signal peptide, *Tb*RRP6ΔC (residues 20–540) lacks the C-terminal region shown to be responsible for the interaction with the exosome complex in yeast [7], [18] but keeps the N-terminal PMC2NT domain, and *Tb*RRP6CAT (residues 176–540) that retains the EXO and HRDC domains responsible for the exoribonuclease activity (Figure 2A). Attempts to express the single recombinant *Tb*RRP6Δsig and *Tb*RRP6ΔC variants failed and the crystallization and catalytic assays were performed only with the *Tb*RRP6CAT variant. However, we were able to co-express both *Tb*RRP6Δsig and *Tb*RRP6ΔC with the *Tb*EAP3 constructions. Recombinant production of the complex *Tb*RRP6Δsig-EAP3 resulted in very low yield of only partially purified products despite our efforts to optimize the expression and purification conditions. In contrast, *Tb*RRP6ΔC was co-expressed with *Tb*EAP3, *Tb*EAP3ΔC1 or *Tb*EAP3ΔC2 and the complexes were successfully purified, giving an average yield of 6 mg of the complexes per liter of culture (Figure 2B). The co-purification of the *Tb*EAP3 constructs with *Tb*RRP6ΔC was directed by the introduction of a His-tag fusion in the *Tb*RRP6ΔC construct whereas the *Tb*EAP3 variants did not contain any tag. The complexes first isolated on a Ni-affinity chromatography were very stable during the subsequent analyses, indicating a strong interaction between *Tb*EAP3 and *Tb*RRP6 proteins *in vitro*. However, *Tb*EAP3 itself proved to be unstable even in complex with *Tb*RRP6ΔC, since we observe a degradation band which corresponds to one of the fragments previously identified by limited proteolysis (Figure 2B).

Recent data using co-purification combined with electrophoretic profile quantification and western-blot analyses showed that yeast Rrp47 and the N-terminal region Rrp6 form a heterodimer *in vitro* [43]. To better characterize the interaction between *T. brucei* RRP6 and EAP3 the complexes *Tb*RRP6ΔC-EAP3ΔC1 and *Tb*RRP6ΔC-EAP3ΔC2 were submitted to SEC-MALS analyses. The molecular masses were estimated in 75.2 kDa and 71.1 kDa for the complexes *Tb*RRP6ΔC-EAP3ΔC1 and *Tb*RRP6ΔC-EAP3ΔC2, respectively, which correspond to heterodimers with stoichiometry of 1∶1 (Figure 2C). The absence of additional peaks corresponding to the individual proteins indicates the high stability of the complex. This result was confirmed by native mass spectrometry (data not shown) and is in accordance with the description of the complex in yeast. The oligomeric state of the *Tb*RRP6CAT variant was also analyzed by SEC-MALS. Its estimated molecular mass was 40.2 kDa which is consistent with the theoretical mass of a monomer (Figure 2C).

Secondary structure content and thermal stability of *Tb*RRP6CAT and the complexes *Tb*RRP6ΔC-EAP3ΔC1 and *Tb*RRP6ΔC-EAP3ΔC2 were investigated using SRCD. The melting temperatures, calculated at 222 nm, and the estimated secondary structure contents are listed in Table 2. The *Tb*RRP6CAT variant was revealed to be very sensitive to temperature increase, showing a calculated T_m_ of approximately 35°C. The melting temperatures of the complexes *Tb*RRP6ΔC-EAP3ΔC1 and *Tb*RRP6ΔC-EAP3ΔC2 were 50°C and 40°C, respectively (Table 2). The difference observed between the complexes suggests that the C-terminal region of *Tb*EAP3ΔC1, that is lacking in *Tb*RRP6ΔC-EAP3ΔC2, may have a role in the complex stability. Because of the importance of divalent metals for RRP6 activity, we tested the effect of magnesium on its folding and stability. No significant differences in melting temperatures or in secondary structure contents were observed for *Tb*RRP6CAT and for the *Tb*RRP6ΔC-EAP3ΔC1 complex upon addition of 5 mM of MgCl_2_ (data not shown).

### The Crystal Structure of the Catalytic Core of *Tb*RRP6

**Figure 3:**
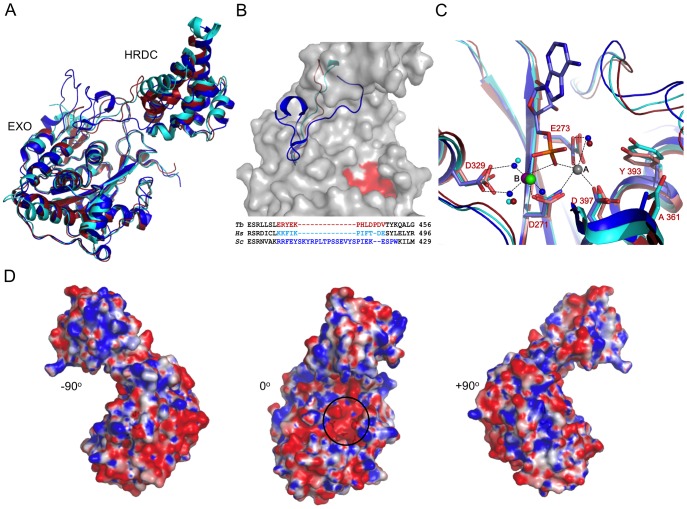
Structure of *T. brucei* RRP6 catalytic core. A) Overall structural comparison of the catalytic core of apo *T. brucei* RRP6 (red), *H. sapiens* RRP6 (cyan) (PDB code 3SAF) and *S. cerevisiae* Rrp6 (dark blue) (PDB code 2HBL). EXO and HRDC domains are indicated. B) Structural comparison of the linker region between the EXO and HRDC domains of *T. brucei*, *H. sapiens* and *S. cerevisiae* RRP6 proteins. The linkers are colored as in (A). The molecular surface of *Tb*RRP6 is shown in gray with the active site residues highlighted in red. A structure-based sequence alignment is shown at the bottom of the picture. C) DEDD-Y active site of apo *Tb*RRP6 (red) superposed to the Mg-bound *Hs*RRP6-D313N mutant (cyan) and *Sc*Rrp6-Y361A mutant (dark blue) bound to one AMP, a zinc and a manganese ion. Water molecules are represented in the same color as the protein. Manganese and zinc (*Sc*Rrp6 structure) are represented in purple (metal B) and gray (metal A) and magnesium (*Hs*RRP6 structure) is represented in green. Residues numbers correspond to the *T. brucei* structure. *Sc*Rrp6 active site interactions are indicated by dotted lines. We observe that *Tb*RRP6 conserves a water molecule in the position of the hydrolytic water that interacts with Y393. Alanine residues (A361 in *Tb*RRP6) replace the aspartate D404 of *Hs*RRP6 in *T. brucei* and yeast. D) Electrostatic surface of the *Tb*RRP6 catalytic core. The bounds for potential contour map visualization are +/−5 kT/e. The active site cavity is indicated with a black circle.

Despite extensive trials, we were not able to crystallize any of the *Tb*EAP3 variants or the complexes *Tb*RRP6ΔC-EAP3ΔC1 and *Tb*RRP6ΔC-EAP3ΔC2. The *Tb*RRP6CAT construct crystallized in a sea urchin-like form composed of very thin needles. The crystals showed to be very hard to reproduce and to optimize and a single data set could be collected from an eventual protuberant needle. *Tb*RRP6CAT crystal structure was refined at 2.4 Å resolution to final R_factor_/R_free_ of 16%/22%, respectively (Table 1). The model covers residues 176 to 541 and includes 185 solvent molecules. The polypeptide chain was clearly defined by the electron density, except the residues 416 to 423 that could not be modeled. The 3D structure of the RRP6 catalytic domain was previously described for the yeast and human counterparts [20], [21]. *Tb*RRP6CAT shares 41% and 40% of sequence identity with the corresponding catalytic core of the yeast and human proteins respectively and, as expected, conserves their overall architecture. The EXO domain consists of a classical α/β fold composed by a six-stranded β-sheet flanked by α-helices and the HRDC domain is constituted of six α-helices (Figure 3A). Superposition of the *T. brucei* RRP6 structure with the human and yeast orthologues results in RMSD of 1.38 Å for 343 C-alpha atoms aligned and 1.42 Å for 333 C-alpha aligned, respectively. As previously described [20], [21] the EXO and HRDC domains are connected by a linker. Comparison of the structures of the yeast and human orthologues showed that the longer linker of yeast Rrp6 narrows the active site entrance which was proposed to affect the ability of the yeast enzyme to degrade structured RNA substrates [21]. The *T. brucei* RRP6 structure shows a closer match to the human RRP6, both proteins presenting a shorter linker and a more accessible active site (Figure 3B).

*Tb*RRP6CAT is the first RRP6 described up to now that was crystallized with the native catalytic site DEDD-Y comprised by the residues D271, E273, D329, D397 and Y393. In the *Tb*RRP6CAT crystal structure, these residues exhibit a configuration similar to the mutated active sites of the human and the yeast structures, even in the absence of metal ions or nucleotides (Figure 3C). The apo *Tb*RRP6CAT catalytic site shows only moderate movements of the side chains comparing with Mn/AMP bound Rrp6 structure, and a water molecule that is coordinated by Y393 and E273 residues could represent the hydrolytic water (Figure 3C). The aspartate D404 of the human RRP6, proposed to have a role in modulating the enzyme activity [21], is replaced by alanine in both yeast and *T. brucei*.

The electron density map revealed the presence of a disulfide bond between the residues C496 and C515, linking α-helix 3 and the end of α-helix 4 of the HRDC domain (Figure S2). C515 is conserved in kinetoplastids and in human RRP6 but it is not present in the yeast orthologue. In contrast, C496 is not conserved among the kinetoplastid homologues. In order to study the effect of the disulfide bond disruption on the protein structure and activity, site directed mutations were introduced in the cysteine residues generating the variants *Tb*RRP6CAT-C496S and *Tb*RRP6CAT-C595S. Crystallization trials were performed and crystals suitable for X-ray diffraction experiments were obtained for the mutant *Tb*RRP6CAT-C496S. A complete data set was collected and the *Tb*RRP6CAT-C496S structure was refined at 2.15 Å resolution to final R_factor_/R_free_ of 17%/22% (Table 1). The model covers residues 179 to 540 and includes 212 waters and 2 PEG molecules. Similarly to *Tb*RRP6CAT the residues 416 to 423 could not be modeled. Superposition of *Tb*RRP6CAT and the mutant *Tb*RRP6CAT-C496S structures resulted in an RMSD of 0.51 Å for 352 C-alpha atoms aligned. The structural changes resulted from the disulfide bond disruption are restricted to one of the helices involved in the SS bond. In the oxidized protein, the short turn that follows helix 4 moves away from helix 3 for the SS bond formation. An investigation of a putative role of the disulfide bond in the *Tb*RRP6 activity is presented in the next paragraph.

### *Tb*RRP6 is more Efficient in the Presence of Manganese Ion and SS Bond Disruption does not Affect RNA Degradation Activity in vitro

**Figure 4:**
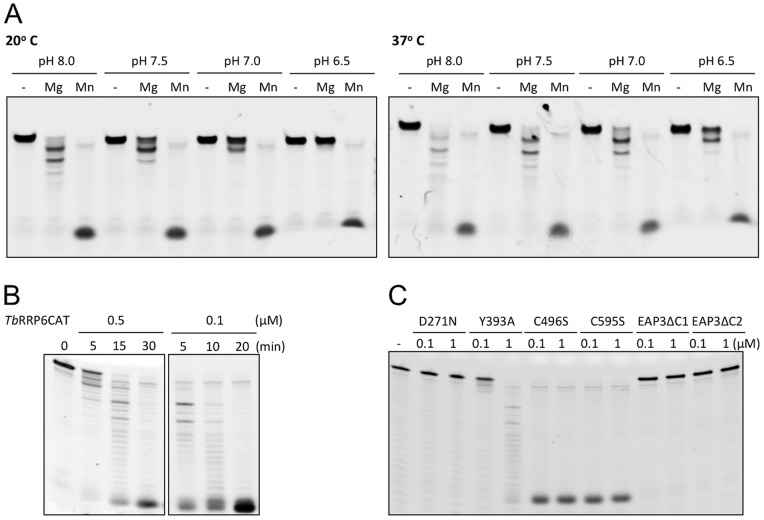
Exoribonucleolytic activity of *Tb*RRP6 under different biochemical and temperature conditions. All assays were performed with 0.1 µM of a 30-mer single-stranded RNA substrate (ssRNA, see materials and methods). A) Single-point RNA degradation assay using *Tb*RRP6CAT at 0.5 µM and incubation of 40 minutes. The reactions were performed in absence (−) and in presence of manganese (Mn) or magnesium (Mg) salts and in different pH and temperatures, as indicated at the top of the gels. B) Time course assay in the presence of magnesium (left) or manganese (right) ions. Enzyme concentration and incubation times are indicated at the top of the gel. We observe faster RNA degradation in the presence of manganese even at lower protein concentration. C) Exoribonucleolytic activity tests of the mutants *Tb*RRP6CAT-D271N, *Tb*RRP6CAT-Y393A, *Tb*RRP6CAT-C496S, *Tb*RRP6CAT-C595S. Assays were conducted at two protein concentrations, as indicated at the top of the gel. The first lane corresponds to the reaction mixture without protein (−) and *Tb*EAP3 mutants which were not expected to present ribonucleolytic activity were also used as negative controls.

The exoribonucleolytic activity of *Tb*RRP6CAT was initially tested in single-point RNA degradation assays. Reactions were performed at different pH and temperatures, using either magnesium or manganese as cofactor. A 30-mer single-stranded 5′-fluorescein labeled RNA (ssRNA, see material and methods) was used as substrate. As expected, no degradation activity was observed when the reaction mixtures do not contain any divalent metal. On the other hand, a significant increase in RNA degradation efficiency is observed when manganese ion is used instead of magnesium (Figure 4A). In addition, we observe that *Tb*RRP6CAT conserves the catalytic activity in the range of temperature (20–37°C) and pH (6.5–8.0) tested (Figure 4A). In the presence of manganese, *Tb*RRP6CAT degraded the RNA substrate completely under all the conditions assayed. However, in the presence of magnesium *Tb*RRP6CAT is more efficient at 37°C and pH 8.0 (Figure 4A). To confirm the *Tb*RRP6CAT preference for the manganese ion, time course assays were performed which indicated that *Tb*RRP6CAT is at least five times more efficient in the presence of manganese as compared to the same reaction in the presence of magnesium (Figure 4B).

The exoribonucleolytic activity of the point mutants *Tb*RRP6CAT-C496S, *Tb*RRP6CAT-C595S, *Tb*RRP6CAT-D271N and *Tb*RRP6CAT-Y393A were also assayed. As previously observed for yeast and human RRP6 proteins [21], the active site mutation D271N abolishes activity, while the mutant Y393A retains activity although the degradation efficiency is highly compromised. On the other hand, the mutants C496S and C595S showed activity comparable with the native protein, indicating that the disruption of the SS bond in the HRDC domain does not affect *Tb*RRP6 degradation of non-structured substrates *in vitro* (Figure 4C). The variants *Tb*EAP3ΔC1 and *Tb*EAP3ΔC2, which are not expected to present ribonucleolytic activity, were also assayed as negative controls, to verify that our purification protocol is efficient to eliminate any RNase activity from residual bacterial contaminants.

### *Tb*RRP6 is able to Degrade Double-stranded and Structured RNA Substrates without a 3′ Overhang

**Figure 5:**
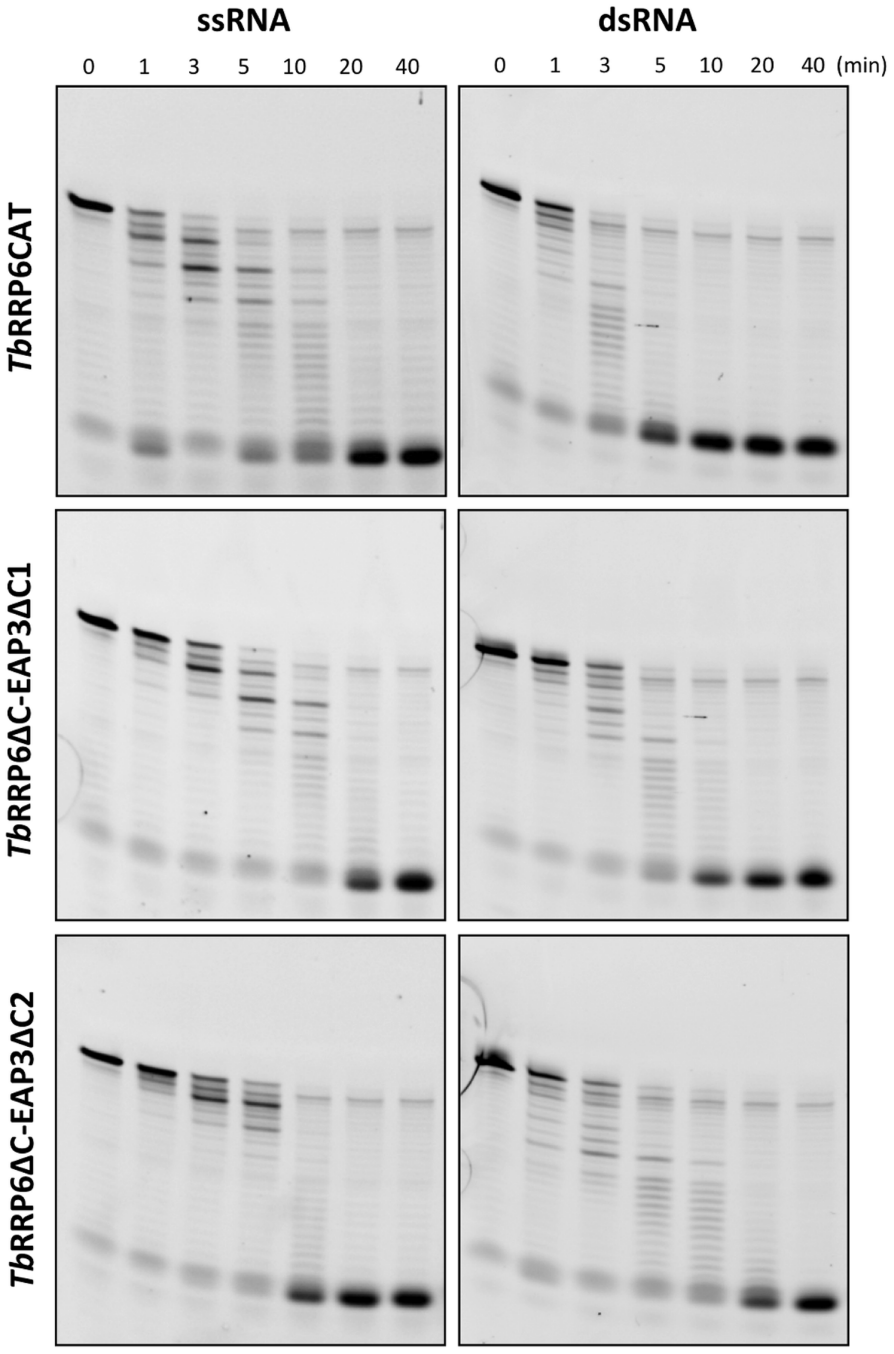
Exoribonucleolytic activity on single-stranded (ssRNA) and double-stranded (dsRNA) RNA substrates. Time-course degradation assays were performed with substrates and protein concentration of 0.1 µM. Substrates and reaction time points are indicated at the top of the gels and the proteins/complexes are identified on the left.

**Figure 6:**
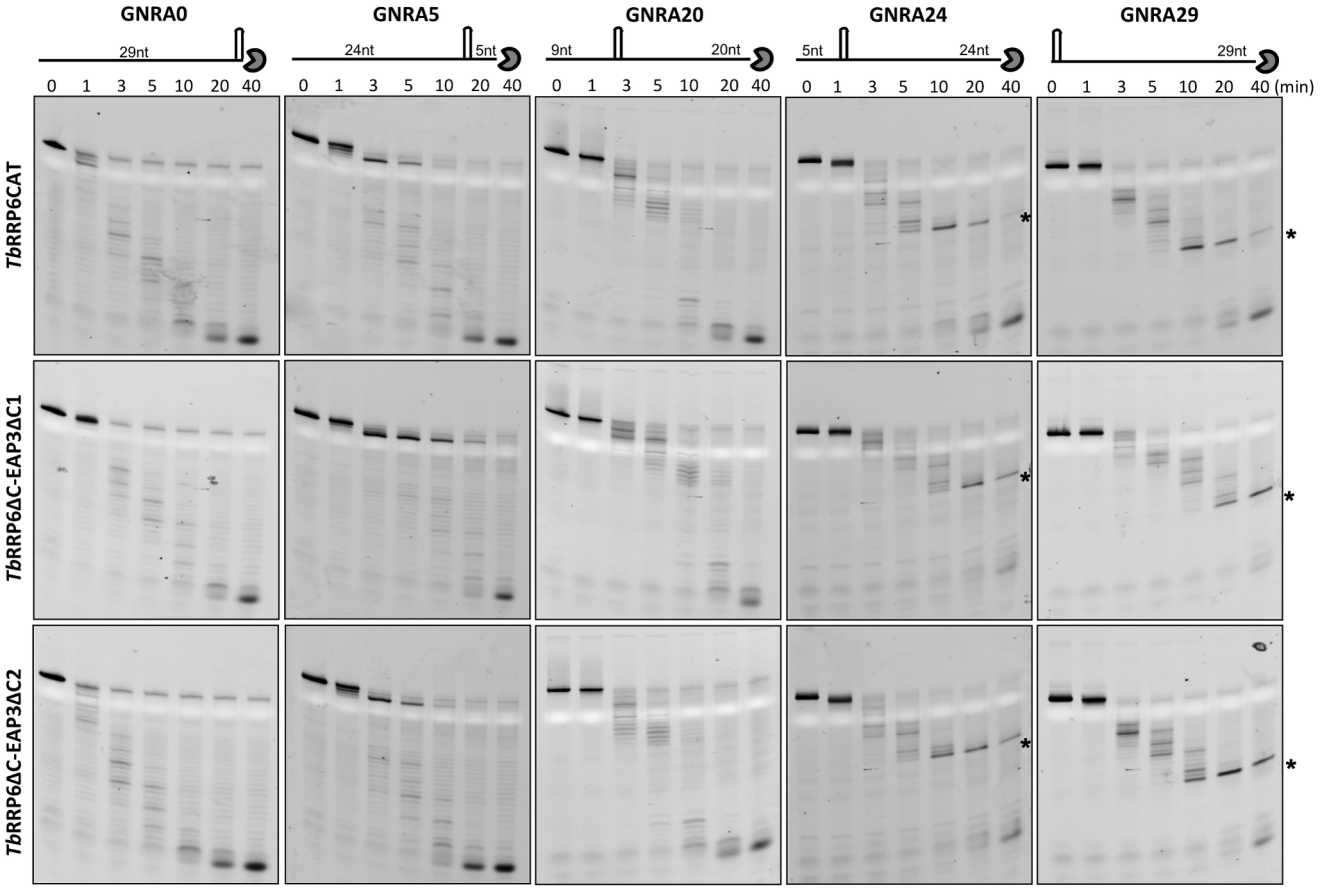
Exoribonucleolytic activity on structured RNA substrates. Time-course degradation assays were performed with protein concentration of 0.1 µM and 0.1 µM of the AU-rich substrate containing a GNRA stem-loop at different positions of the strand (see material and methods). The substrates are named accordingly to the size of the 3′ single-stranded extension, as schematically indicated at the top of the gels. The reaction time points are shown in minutes. The asterisks indicate the most stable intermediates observed during degradation.

Time-course degradation assays were performed with *Tb*RRP6CAT variant and complexes *Tb*RRP6ΔC-EAP3ΔC1 and *Tb*RRP6ΔC-EAP3ΔC2 using different synthetic RNA substrates. Initially, protein activity was tested against a 30-mer single-stranded RNA, and the detection of decreasing size intermediates prior to accumulation of the final product indicates the distributive exoribonucleolytic activity of *Tb*RRP6 (Figure 5, left), similarly to results previously described for yeast and human orthologues [21]. Moreover, our results show that upon association with EAP3, although the activity slows as evidenced by the degradation pattern after 1 and 3 minutes, the complex is still able to degrade the non-structured RNA substrate completely. More surprising is the observation that *Tb*RRP6CAT and the *Tb*RRP6ΔC-EAP3ΔC1 and *Tb*RRP6ΔC-EAP3ΔC2 complexes also degrade the double-stranded RNA substrate efficiently (Figure 5, right). The mutants *Tb*RRP6CAT-C496S and *Tb*RRP6CAT-C595S, lacking the SS bond, were also assayed against double-stranded RNA and showed activity comparable with the constructs with native cysteine residues (Figure S3). Previous studies reported that the yeast and human ribonucleases RRP44 and RRP6 require a 3′ single-stranded extension to start the substrate degradation [6], [10], [21]. By contrast, our results show that *T. brucei* RRP6 is able to degrade double-stranded RNA without any 3′ overhang. To further evaluate the activity of *T. brucei* RRP6 against structured RNA substrates and to compare with previous results obtained for yeast and human enzymes [21], a set of synthetic RNAs were designed containing a GNRA stem-loop in different positions of an AU-rich chain. The substrates were named GNRA0, GNRA5, GNRA20, GNRA24 and GNRA29, where the ending number indicates the number of nucleotides of the 3′ single strand. Time-course degradation assays were performed with *Tb*RRP6CAT and the *Tb*RRP6ΔC-EAP3ΔC1 and *Tb*RRP6ΔC-EAP3ΔC2 complexes. Again, no significant difference was observed between the activity of *Tb*RRP6CAT and the complexes (Figure 6). Intermediates resistant to degradation are present when the secondary structure is positioned close to the 5′ end (GNRA24 and GNRA29), indicating that a 5′ overhang is needed for efficient activity, as previously described for the yeast and human orthologues [21]. It has been suggested that the HRDC domain would interact with the 5′ single strand, stabilizing the binding to the substrate long enough to allow degradation [21]. In contrast, our results show that *Tb*RRP6 is able to degrade 3′ double-stranded RNA and RNA substrates containing stem-loops at the 3′-end without any overhang (GNRA0) (Figure 6). To our knowledge this is the first time that such an activity is reported for an RRP6 orthologue.

## Discussion

*T. brucei* RRP6 was previously characterized as an essential structural subunit of the exosome complex found both in the nucleus and in the cytoplasm [26], [27], distinguishing the trypanosome exosome from those of humans and yeast. In this work, we aimed to obtain functional information on *T. brucei* RRP6 and data on its substrate preferences and regulation by the putative interacting partner *Tb*EAP3. We succeeded in determining the crystal structure of the catalytic domain of *Tb*RRP6 containing the native catalytic site residues, which is a novel result as compared to the yeast and human RRP6 orthologues whose structures were determined using inactive catalytic site mutants. Another important novel finding was the fact that *Tb*RRP6 is able to degrade both 3′ double-stranded RNA substrates and 3′-end structured RNA. Furthermore, we reconstituted several *Tb*RRP6-*Tb*EAP3 complex variants and performed RNA degradation assays showing that there is no detectable effect of *Tb*EAP3 interaction with *Tb*RRP6 on RNA degradation *in vitro* under the conditions tested in this work. The heterodimer formed by *Tb*EAP3 and *Tb*RRP6 *in vitro* is consistent with the interaction detected between these proteins by two-hybrid analysis [26] and suggests that *Tb*EAP3 belongs to the functional Rrp47/C1D protein family despite their primary structure divergence. We have also shown that recombinant *Tb*EAP3 is a homodimer in solution. The biological relevance of the oligomer of *Tb*EAP3 and whether the *Tb*EAP3 homodimer is dissociated to allow the formation of the heterodimer with *Tb*RRP6 in* vivo* remain to be determined. Nevertheless, the biochemical behavior of the recombinant *Tb*EAP3 could be an indication of its role as a platform for protein interaction and binding of nucleic acid substrates given its propensity to form aggregates and its instability in solution.

It was previously reported that the native exosome purified from *Leishmania tarentolae* lacked the hydrolytic RNase-D like activity usually attributed to the RRP6 subunit [28]. The explanation proposed for this finding suggested that kinetoplastidae RRP6 could be inactive, the substrates tested were inappropriate or that the association of RRP6 with the exosome or with EAP3 could regulate RRP6 activity [28]. More recently, accumulation of mRNA degradation intermediates containing 5′ ends was described for *T. brucei* with impaired exosome activity, suggesting that a distributive 3′–5′ exoribonuclease activity should be present in trypanosome [44]. In this work, we show that *Tb*RRP6 does have distributive 3′–5′ exoribonuclease activity *in vitro*. Moreover, association with EAP3 did not significantly affect degradation activity of *Tb*RRP6ΔC on the substrates tested (Figures 5 and 6). Studies in yeast cells have indicated that Rrp47 functions together with Rrp6 for selection of some RNA substrates to be degraded [22]. Thus, although we have not detected a significant effect of *Tb*EAP3 association on the *Tb*RRP6 activity towards the synthetic substrates used in this work we do not exclude that a regulation might occur *in vivo*. We also hypothesize that the C-terminal truncations of the *Tb*EAP3 variants could have affected the RNA binding properties of EAP3 and its ability to modulate *Tb*RRP6 activity.

The crystal structure of the *Tb*RRP6 EXO-HRDC catalytic core revealed the native catalytic site DEDD-Y which retains the conformation of the ion/nucleotide bound active sites described for the RRP6 mutants from human and yeast orthologues. We have also shown that *Tb*RRP6 is more efficient in degrading RNA substrates *in vitro* in the presence of manganese instead of magnesium, consistent with the results of Midtgaard and co-workers which suggest that manganese and zinc are specifically required in the active site of Rrp6 [20]. However, the rationale behind the divalent metal ion preferences among the DEDD nucleases is unclear. Structural comparison of apo and holo active sites of *T. brucei*, yeast and human RRP6 and *E. coli* RNase D, which is more efficient in the presence of magnesium [45], did not allow us to propose a mechanistic basis for the differing effectiveness of the metal ions in catalysis, since the structure of the active sites is highly conserved (not shown).

To our knowledge, the ability of *Tb*RRP6 to degrade double-stranded and 3′-end structured RNA substrates is a new finding for this class of DEDD 3′–5′exonucleases. Degradation of structured RNA/DNA requires an initial step of substrate unwinding before cleavage can occur. Some nucleases are able to degrade structured substrates in the absence of other factors, while others are dependent on association with helicases for this function. RRP6 contains a HRDC (helicase and RNAseD C-terminal) domain but the function of this domain is not completely elucidated. Previous studies revealed that removal of the HRDC domain in yeast Rrp6 disrupts the processing of certain RNA substrates *in vivo* [18]. The recombinant human RRP6 protein lacking the HRDC domain exhibited 100-fold reduction in exoribonuclease activity [21], and disruption of the exonuclease-HRDC domain contact was proposed to prevent proper orientation of certain substrates in the active site [20]. Also, it was previously reported that non-catalytic basic amino acids cooperatively contribute to destabilize double-stranded RNA to degradation by single-strand-preferring ribonucleases [46]. The electrostatic surface of *Tb*RRP6 (Figure 3D) evidences positively charged regions surrounding the active site and a basic path at one side of the HRDC domain. Moreover, analysis of the *Tb*RRP6CAT structure shows basic residues located on the periphery of the catalytic site, most of them conserved in yeast and human orthologues (Figure S4). Further assays are necessary to evaluate the role of the HRDC domain and of individual basic residues in the ability of *Tb*RRP6 to degrade 3′-end structured RNA, but we speculate that the HRDC domain may function to correctly orientate the substrates, and active site peripheral basic residues may contribute to the destabilization of the double-strand and preparation of the 3′-end for cleavage.

Finally, the differences described for the *T.brucei* exosome compared with the human and yeast complexes, notably, the association of *Tb*RRP6 to both nuclear and cytoplasmic exosomes in stoichiometric amounts and the absence of a Rrp44-like protein in *T. brucei* purified exosome fractions [26], [27], could indicate that RRP6 may have a more extensive role in the RNA processing and degradation pathways in trypanosomes, which could be related to its ability to degrade double-stranded and 3′-end structured RNA substrates.

## Supporting Information

Figure S1 **SRCD spectra showing the thermal denaturation of the** ***Tb*** **EAP3ΔC1 variant.** Temperature scans (shown in rainbow colors) were performed between 18°C and 78°C, using 3°C steps with 2 minutes settling time.
(TIF)

Figure S2 **Exoribonucleolytic activity of the mutants** ***Tb*** **RRP6CAT-C496S and** ***Tb*** **RRP6CAT-C595S on single-stranded (ssRNA) and double-stranded RNA (dsRNA).** Time-course degradation assays were performed with substrates and protein concentration of 0.1 µM. Substrates and reaction time points are indicated at the top of the gels and the proteins are identified on the left. Comparing with *Tb*RRP6CAT protein (Figure 5) no significant difference in activity was detected.
(TIF)

Figure S3 **Structural comparison of the HRDC domains of** ***Tb*** **RRP6CAT (red) and** ***Tb*** **RRP6CAT-C496S mutant (yellow).** The disulfide bond is coloured in green. On the right, 2Fo-Fc electron density maps of native (top) and mutant (bottom) proteins are shown in gray and contoured at 1.2 σ.
(TIF)

Figure S4 **Basic residues surrounding** ***T. brucei*** **RRP6 active site.** *Tb*RRP6CAT surface is represented in gray with arginine and lysine residues highlighted in blue. The active site cavity is coloured in red. *Tb*RRP6 residues K374, K335 and R439 are conserved in human (K417, K377 and K480, respectively) and yeast (K342, R302 and R400, respectively) orthologues; K374 is conserved in yeast Rrp6 (K343). The residues R327 and R331 are not conserved in the human and yeast sequences but the side chains of K479 and R399 (human and yeast, respectively) are orientated in such a way as to occupy similar positions to the *Tb*RRP6CAT side chains.
(TIF)

Table S1 **Primers used for site-directed mutagenesis.** Mutated bases are represented in red.
(DOCX)
