# Supplementary figures and images for: RRP6 from Trypanosoma brucei: Crystal Structure of the Catalytic Domain, Association with EAP3 and Activity towards Structured and Non-Structured RNA Substrates
Source: PLoS One. 2014 Feb 18;9(2):e89138. doi: 10.1371/journal.pone.0089138 (PMC3928423; doi:10.1371/journal.pone.0089138)

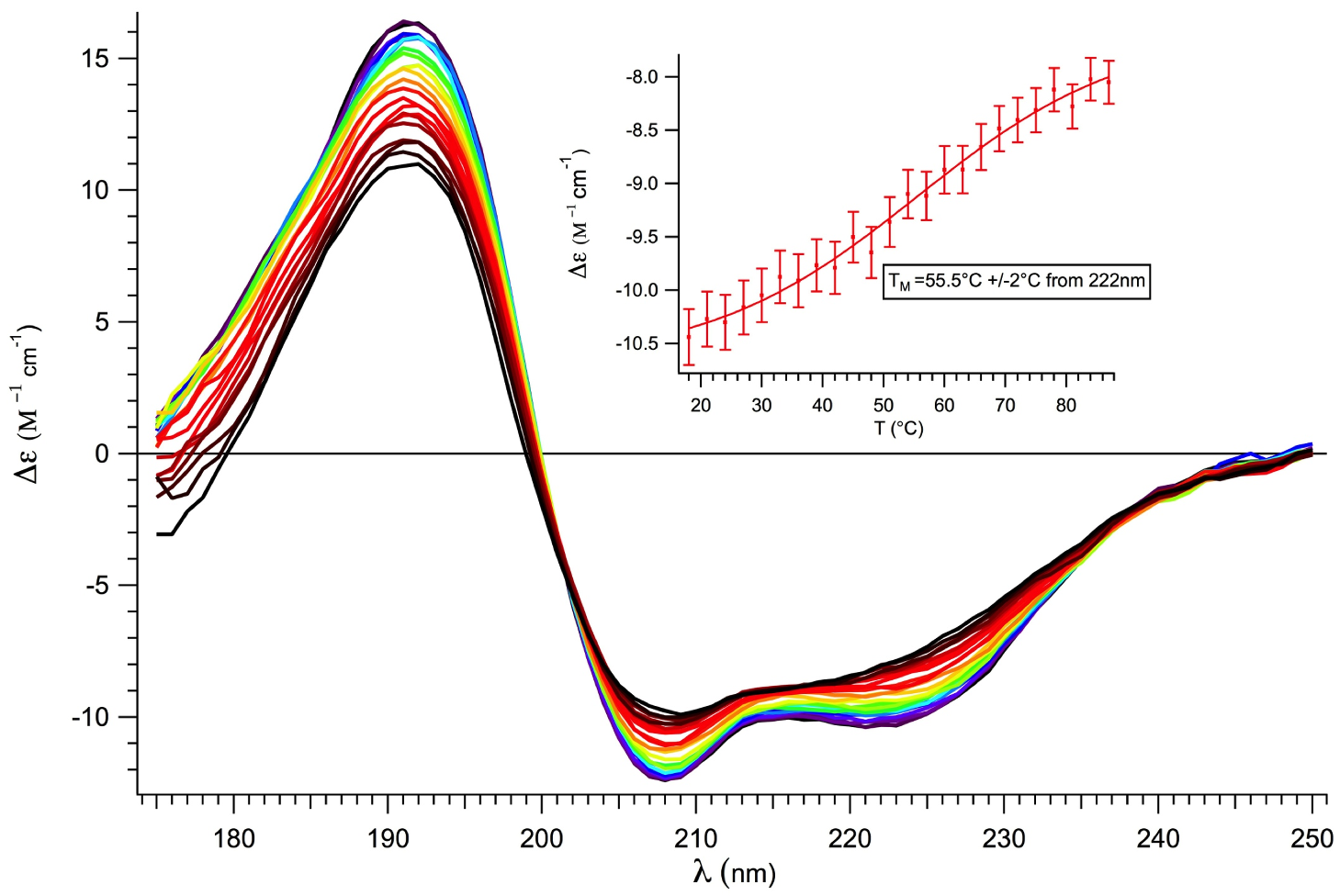

Supplement: Figure S1 — SRCD spectra showing the thermal denaturation of the Tb EAP3ΔC1 variant. Temperature scans (shown in rainbow colors) were performed between 18°C and 78°C, using 3°C steps with 2 minutes settling time. (TIF) [file pone.0089138.s001.tif]

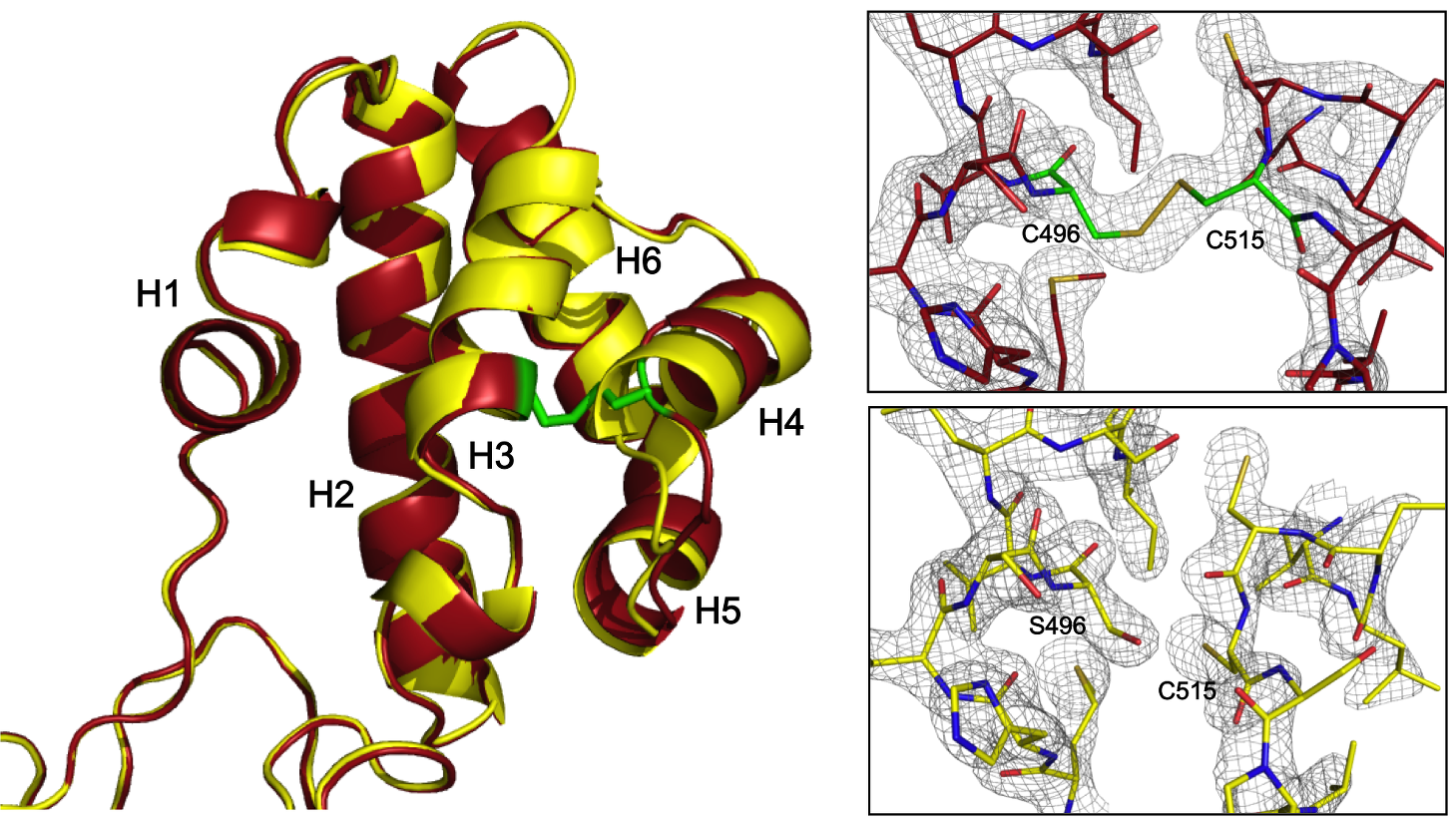

Supplement: Figure S3 — Structural comparison of the HRDC domains of Tb RRP6CAT (red) and Tb RRP6CAT-C496S mutant (yellow). The disulfide bond is coloured in green. On the right, 2Fo-Fc electron density maps of native (top) and mutant (bottom) proteins are shown in gray and contoured at 1.2 σ. (TIF) [file pone.0089138.s003.tif]

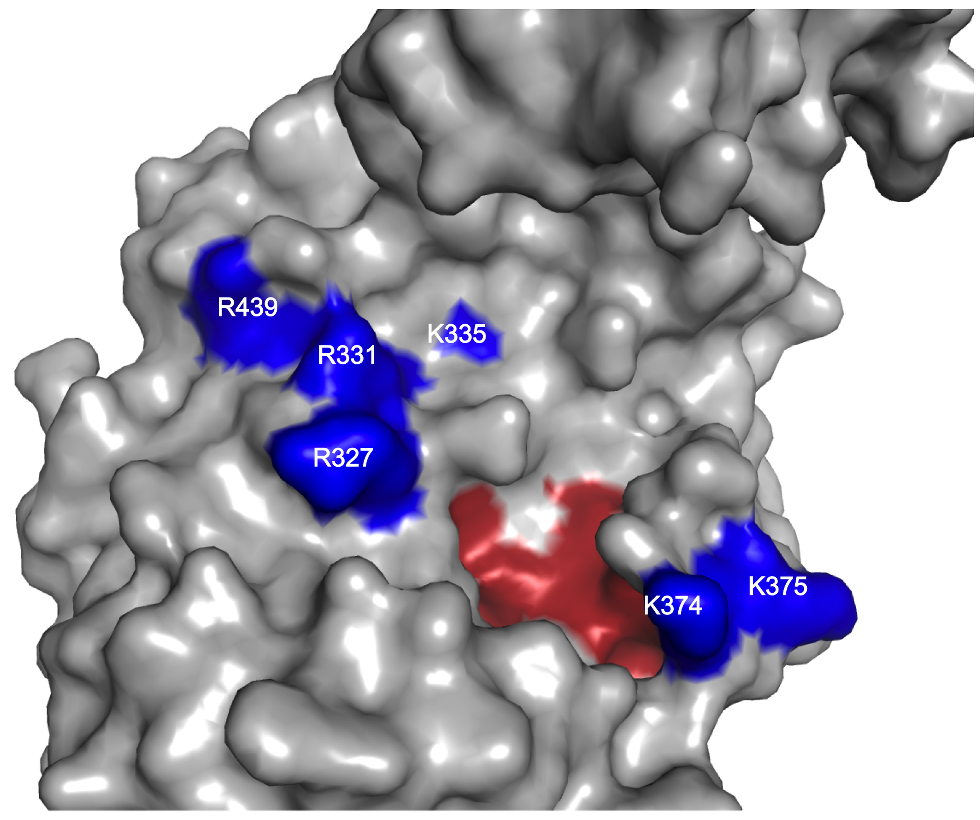

Supplement: Figure S4 — Basic residues surrounding T. brucei RRP6 active site. TbRRP6CAT surface is represented in gray with arginine and lysine residues highlighted in blue. The active site cavity is coloured in red. TbRRP6 residues K374, K335 and R439 are conserved in human (K417, K377 and K480, respectively) and yeast (K342, R302 and R400, respectively) orthologues; K374 is conserved in yeast Rrp6 (K343). The residues R327 and R331 are not conserved in the human and yeast sequences but the side chains of K479 and R399 (human and yeast, respectively) are orientated in such a way as to occupy similar positions to the TbRRP6CAT side chains. (TIF) [file pone.0089138.s004.tif]
