# Supplementary material for: RRP6 from Trypanosoma brucei: Crystal Structure of the Catalytic Domain, Association with EAP3 and Activity towards Structured and Non-Structured RNA Substrates
Source: PLoS One. 2014 Feb 18;9(2):e89138. doi: 10.1371/journal.pone.0089138 (PMC3928423; doi:10.1371/journal.pone.0089138)
Supplement: Table S1 — Primers used for site-directed mutagenesis. Mutated bases are represented in red. (DOCX) [file pone.0089138.s005.docx]

**Table S1:** Primers used for site-directed mutagenesis. Mutated bases are represented in red.

| **Primer** | **Sequence** |
| --- | --- |
| D271N-f | 5’-gaaaccgaaattgcagttaatctggaacaccacgattt-3’ |
| D217N-r | 5’-Aaatcgtggtgttccagattaactgcaatttcggtttc-3’ |
| Y393A-f | 5’-cagaaatggttagcgctgcacagcaggatacc-3’ |
| Y393A-r | 5’-Ggtatcctgctgtgcagcgctaaccatttctg-3’ |
| C496S-f | 5’-cagttatgcatattagctctgttctgagcattgcaacc-3’ |
| C496S-r | 5’-Ggttgcaatgctcagaacagagctaatatgcataactg-3’ |
| C515S-f | 5’-tgaagttctgaaatgttctagtccggttagcgttgc-3’ |
| C515S-r | 5’-Gcaacgctaaccggactagaacatttcagaacttca-3’ |
